# Let Nature Remove the Placenta

**Published:** 1884-05

**Authors:** 


### LET NATURE REMOVE THE PLACENTA.

In the *Deutsche Medical Wochenschrift*, Dr. Dohrn thus sums up his experience: "1. In one thousand lying-in women, in whom the expulsion of the placenta was left to nature, the results were far better than in one thousand others in whom Credé's method of expulsion was used. 2. The one thousand lying-in women in whom the placenta was spontaneously expelled had considerable less hæmorrhage and fever after delivery. In those cases treated by Credé's method portions of the membranes were frequently retained, and there were more fatal cases than in the others. 3. The disadvantages which are conditional to the method of Credé are especially seen in the cases in which the placenta is expressed during the first five minutes. After a longer time the expression was more complete, but never as safe as by the spontaneous method."

